# Assessing the thyroid autoimmunity association with recurrent aphthous stomatitis

**DOI:** 10.1186/s12903-023-03326-y

**Published:** 2023-08-30

**Authors:** Fariba Karimi, Fatemeh Lavaee, Aida Nematollahi, Sarina Sahmeddini

**Affiliations:** 1 Endocrinology and Metabolism Research center, Shiraz University of Medical Sciences, Shiraz, Iran; 2 Oral and Dental Disease Research Center, Shiraz University of Medical Sciences, Oral and Maxillofacial Disease Department, School of Dentistry, Shiraz, Iran; 3 Student Research Committee, Shiraz University of Medical Sciences, Shiraz, Iran; 4 Department of Microbiology, The Forsyth Institute, Cambridge, MA USA

**Keywords:** Aphthous stomatitis, recurrent, Autoimmune diseases, Thyroid diseases

## Abstract

**Background:**

Recent investigations have highlighted autoimmune origins and abnormal immune responses; particularly those related to T cell-mediated immunity and elevated T lymphocyte cells in the oral mucosa. Therefore, we investigated the relationship between recurrent aphthous stomatitis (RAS) and autoimmune thyroid diseases (ATDs) in an Iranian population.

**Methods:**

A cross-sectional study was performed on 102 patients diagnosed with ATD (cases) and 102 healthy patients (controls) who had been referred for the routine dental treatment. All participants were asked for the history of RAS and their age, gender, other systemic diseases, medications, and frequency of RAS in a year. Matching was performed based on the propensity scores for age and sex. In addition, the number of lesions in each recurrence in both groups was assessed and compared. The type of thyroid disease has been assessed for case participants and has been confirmed by the endocrinologist. The chi-square test, t-test, and Mann-Whitney U test were used to analyze the data using SPSS 18.

**Results:**

Patients with ATD had higher RAS than healthy controls (P = 0.040). ATD patients had 1.93 times more risk for RAS, and the frequency of RAS in a year was 3.15times higher in these patients (P = 0.011). Moreover, the frequency of RAS was higher in patients with hypothyroidism than in those with hyperthyroidism. However, there were no significant differences in the size and the number of lesions between the groups.

**Conclusion:**

The risk and frequency of RAS were significantly higher in patients with ATDs. This would provide valuable insights into the underlying mechanisms and potential treatment strategies for both conditions.

**Supplementary Information:**

The online version contains supplementary material available at 10.1186/s12903-023-03326-y.

## Introduction

Recurrent aphthous stomatitis (RAS) is characterized by single or multiple rounded ulcers [1]. RAS ulcers may exceed 1 centimeter and take 30 days to heal; as a result, they can be painful, disturb eating and speaking, and reduce quality of life [2]. Although there are many local, systemic, genetic, and immunopathogenic factors associated with RAS, the exact etiology underlying RAS remains unclear [3].

Recently, many investigations have focused on autoimmune origins and abnormal immunological responses. Immune dysfunction is found to be associated with T-cell-mediated immunity, which causes the elevation of T-lymphocyte cells. Also, lymphocyte cytotoxicity and antibody-dependent cell-mediated cytotoxicity in the oral mucosa are the most important factors. Therefore, it is a rational explanation for the association between the immunological etiology of RAS and autoimmune diseases [4–6].

More recent investigations also showed a relationship between the severity of RAS and CD4 + and CD8 +, CD4 + to CD8 + ratios, and IL2, INF ɣ, also TNFα have been found to be significantly increased in these patients [7]. Moreover, it was found that nearly one-third of RAS patients may have high serum levels of anti-thyroglobulin (TG) and anti-thyroid peroxidase (TPO) [8].

It is now clear that the thyroid gland is a common target of autoimmune disorders. Similar to RAS, the exact mechanism of autoimmune thyroid disease (ATD) has not been completely clarified, but genetic, immune system, and environmental risk factors have been recommended to be important in the pathogenesis of these diseases [9]. ATDs are characterized by antibodies against TG and thyroid stimulating receptors (TSHR); therefore, keratinocytes expressing TSHR and TG can also be the target of these autoantibodies [10]. Therefore, we hypothesized that circulating autoantibodies in patients with ATD can target proteins of the keratinocyte membranes of the oral mucosa, leading to RAS ulcers.

Despite the importance of understanding the association between RAS and ATD as two common diseases, few studies have been conducted. If there is an association between RAS and ATD, the dentist manage RAS more easily. Hence, the aim of the present study was to investigate the relationship between RAS and ATD in an Iranian population.

## Materials and methods

This cross-sectional study was conducted between 2020 and 2021. This study included 102 patients diagnosed with ATD (case), who had been referred to the Emam Reza clinic for receiving treatment, and 102 healthy patients (control), who had been referred for routine dental treatment, including scaling root planning, simple root canal therapies, and restorative procedures, to the dental faculty of Shiraz University of Medical Sciences. Matching was performed based on the propensity score by logistic regression for age and sex.

ATD patients were diagnosed based on their thyroid hormone serum level disturbance and serum anti-TG (radioimmunoassay) and anti-thyroid peroxidase antibody (ATPO, by radioimmunoassay) levels by an endocrinologist. They were not under any treatment for their ATD, and after assessment of the patient’s history, treatment was started.

Pregnant women, patients with other diseases, those taking other medications that can cause oral ulcers, and diabetic patients were excluded. Prior to starting the investigation, the ethics committee of Shiraz University of Medical Sciences approved the study (IR.SUMS.DENTAL.REC.1399.151) and all participants signed a written informed consent form. Following the Helsinki declaration, patient information was confidentially recorded.

All participants were asked about their current and history of RAS and their age, sex, other systemic diseases, medications, and frequency of RAS in a year during an interview. To reduce the probability of bias, only RAS that had been diagnosed by a clinician before was added to our data. In addition, the number of lesions in each recurrence in both groups was assessed and compared. The type of thyroid disease including hyperthyroid and hypothyroid disorders were assessed for the case participants and confirmed by an endocrinologist.

The data were analyzed using SPSS version 18, and the T test was used to compare the frequencies of both groups. The chi-square test was used to assess the age, sex, and RAS frequency of the two groups, and the relationship between the number of RAS lesions in each recurrence. Since the distribution of data was not normal, the relationship between the frequency of RAS in a year and ATD was evaluated using the Man-Whitney test. The odds ratio was used to assess the risk of RAS in patients with ATD. Statistical significance was set at P < 0.05.

## Results

In this study, 102 patients with ATD, with a mean age of 40.45 ± 14.33 years old, and 102 healthy participants, with a mean age of 40.56 ± 14.64 years old, have been enrolled. The mean age of the two groups was not statistically different (P = 0.95). The sex distribution of the participants is reported in Table 1. There was no significant difference between the sex of participants in the case and control groups (P = 0.86). In the case group, 15.7% (16 patients) had hyperthyroid disorders, and 84.3% (86 patients) had hypothyroid disorders.

**Table 1:** Sex distribution of participants

		Number (% within Group)		Total
		Male	Female	
Group	Control	23 (22.5%)	79 (77.5%)	102 (100.0%)
	Case	22 (21.6%)	80 (78.4%)	102 (100.0%)
Total		45 (22%)	159 (78%)	204 (100.0%)

The frequency of RAS in the participants in both groups is shown in Table 2. Patients with ATD had more RAS than the healthy controls (P = 0.040). These patients had 1.93times more risk for RAS [OR 1.93 (95% CI:1.03–3.63)].

**Table 2:** The frequency of RAS in participants of both groups

		History of RAS* Number (% within Group)		Total
		No	Yes	
Group	Control	81 (79.4%)	21 (20.6%)	102 (100.0%)
	Case	68 (66.7%)	34 (33.3%)	102 (100.0%)
Total		179 (76.5%)	55 (23.5%)	204 (100.0%)

* RAS: recurrent aphthous stomatitis

Table 3 shows the relationship between the number of RAS lesions and ATD. RAS lesions were most likely single; however, no significant difference in the number of lesions was found between the two groups (P > 0.05). Lesions were mostly (94.5%) less than 1 cm, and there was no significant difference in size between two groups either (P = 0.28). On the other hand, the frequency of RAS in a year was higher in patients with ATD (P = 0.01) (OR 3.15 (95% CI: 1.31–7.57).

**Table 3:** The relationship between the numbers of RAS lesions with autoimmune thyroid disease

	Single Number (% within Group)	Multiple Number (% within Group)	Total Number (% within Group)	P value
Control	17 (81%)	4 (19%)	21 (100.0%)	P = 0.89
Case	28 (82.4%)	6 (17.6%)	34 (100.0%)	
Total	45 (81.8%)	10 (19.2%)	55 (100.0%)	

## Discussion

According to the results of the study, patients with ATD were 1.93 times more likely to have RAS than healthy controls. Although the initiating cause and etiology of RAS are not clear, there are some studies on the coexistence of RAS and autoimmune diseases [11–13]. In a cohort study of a sample population of 1 million, Lee et al., concluded that RAS*-*like lesions may be an early sign of autoimmune diseases, as they were associated with an increased risk of systemic lupus erythematosus, rheumatoid arthritis, gout, ankylosing spondylitis, and Behçet’s disease [13].

Moreover, similar to our study, other investigations have reported relationships between ATD patients and RAS [13–16]. Najafi et al., reported that T3, TSH, and anti-TG levels were significantly higher in the RAS group in comparison to healthy controls, and ATD frequency was significantly higher in patients with RAS [14]. In another investigation on 20 sex and age-matched healthy volunteers, participants with RAS had higher levels of fT4, anti-TPO, and anti-TG than the control group without RAS [15]. This result is also similar to that of Ozdemir et al., study who reported that thyroid nodules were present in 28.8% of patients with RAS [16]. Furthermore, it has been reported that RAS patients have some degree of iodine deficiency, so iodine mouthwash can have great therapeutic effects on RAS [17, 18]. However, further studies required to confirm this.

Although there is no specific mechanism regarding the relationship between ATD and RAS, many studies have reported increased levels of TNFα, IL2 and IFNɣ and decreased levels of IL4 in RAS patients. Since these cytokines are produced by keratinocytes and activate T cells, patients with ATD also have high levels of IL2, TNFα, and INF-γ. Therefore, cytokines may be the most common immunological factors for RAS and ATD [19, 20].

Our results showed that most of the case groups had hypothyroidism (82.6%,100 patients), whereas others had hyperthyroid disorders. This result is similar to that of Ozdemir and Najafi et al., in which more patients were determined to have hypothyroidism than hyperthyroidism [14, 16]. There is no specific reason reported yet for the relationship between the type of thyroid disorder and RAS, and we assume that this difference is because, generally, the number of patients with hypothyroidism is higher than that of with hyperthyroidism.

In the current study, the number of females was higher than males, which is similar to other studies that suggest that females are at a higher risk of RAS development than males [21, 22]. In addition, females had more ATDs than males due to higher rates of anti-TPO positivity in women [23]. Generally, autoimmune illnesses affect women more frequently than men [24, 25].

According to the results, there were no significant differences in the frequency and number of lesions in the healthy and ATD groups. Artuz et al., found no significant relationship between RAS duration and thyroid hormones, including (fT3), (fT4), TSH, thyroxine, anti-TG, and anti-TPO [15]. In contrast, Ozdemir et al., concluded that the duration of RAS was longer in patients with an ATD than in those without an ATD [16].

The results of the current study strongly support the relationship between ATD and RAS. While hypothyroid disorders were more frequent in evaluated participants, a much larger sample size of patients with hypothyroidism should be considered. Furthermore, relying on the patients’ memory of their history of RAS was a limitation of our study.

It can be suggested that dentists order thyroid tests in patients with RAS. Nevertheless, new matched case-control studies are needed to reveal the relationships between ATD and RAS, especially in terms of the frequency and number of lesions and iodine treatments. Also evaluating the participants in the groups with the same number of patients with hypothyroid and hyperthyroid disorders can reveal the association with RAS more preciously.

In addition, monitoring thyroid hormone levels in patients with RAS is recommended.

## Conclusion

RAS frequency was significantly higher in patients with ATDs. However, the frequency and number of lesions had no relationship with ATD. Understanding the correlation between ATD and RAS can greatly enhance clinical practice by enabling more regular monitoring, facilitating early diagnosis, and establishing preventive protocols for these episodes. This knowledge empowers healthcare professionals to optimize patient care and improve overall outcomes.

### Electronic supplementary material

Below is the link to the electronic supplementary material.

Supplementary Material 1

## Data Availability

The data used to support the findings of this study were supplied by Shiraz University of Medical Sciences under license and so cannot be made freely available. Requests for access to these data should be made to Fatemeh lavaee, fatemeh.lavaee@gmail.com or lavaeef@sums.ac.ir.

## List of abbreviations

RAS: Recurrent aphthous stomatitis
TG: anti-thyroglobulin
TPO: anti-thyroid peroxidase
ATD: autoimmune thyroid diseases
TSHR: thyroid stimulating receptors
ATPO: anti-thyroid peroxidase antibody
